# Remote Changes of Mechanical Stiffness Following Local Stretching or Contraction: A Systematic Review with Meta-Analysis

**DOI:** 10.1186/s40798-026-01030-z

**Published:** 2026-06-02

**Authors:** Laurits Kretschmer, Jan Wilke

**Affiliations:** 1 Bayreuth Center of Sport Science, Department of Neuromotorics and Movement, University of Bayreuth, Universitätsstraße 30, 95447 Bayreuth, Germany; 2 Institute of Sport Sciences, Department of Movement Science, University of Klagenfurt, Klagenfurt, Austria

**Keywords:** Fascia, Myofascial chain, Lengthening, Contraction, Remote, Non-local, Force transmission

## Abstract

**Background:**

Fascia links the skeletal muscles, creating a body-wide network of myofascial continuity. Experimental studies confirmed the mechanical relevance of this architecture as substantial amounts of force can be transmitted across tissue connections. Against this background, it has been suggested that exercise may not only modify local but also distant tissue properties. This systematic review with meta-analysis evaluated the effects of local tissue lengthening and local muscle contraction on the stiffness of remote structures within the same myofascial chain.

**Methods:**

Two investigators performed a systematic literature search using Web of Science, PubMed, and Google Scholar. We included controlled experimental trials comparing the effects of local tissue lengthening (e.g., gastrocnemius stretching) or tensioning (e.g., gastrocnemius contraction) on non-local tissue properties (e.g., stiffness of the plantar fascia or erector spinae). Study quality and risk of bias were examined by means of a modified Downs and Black checklist. Funnel plots and Egger’s tests were used to identify reporting bias. We used robust variance estimation to pool the standardized mean differences between intervention (local stretching or contraction) and control (tissue in neutral, slack position). The GRADE criteria were applied to determine the certainty about the evidence.

**Results:**

A total of 15 studies with mostly high methodological quality (11.3±1.7 points) were identified. Visual inspection of funnel plots and the Egger's test revealed a possible reporting bias for contraction and lengthening. While tissue lengthening enhanced distant tissue stiffness with moderate magnitude (Hedges' g= -0.54, 95%CI -1.03 to -0.05, p=0.04, 12 studies, 42 effect sizes (ES), τ² = 0.42, very low certainty), contraction induced a large non-local stiffness increase (g= -1.25, 95%CI -2.15 to -0.34, p=0.016, 7 studies, 67 ES, τ² = 0.61, moderate certainty).

**Conclusion:**

Both muscle contraction and stretch increase non-local stiffness in structurally connected body regions. This finding is of relevance for exercise professionals and therapists aiming to optimize performance or rehabilitation. The high heterogeneity and considerable differences between the individual study results, particularly regarding local lengthening, call for further research.

*Trial Registration:*Prospective registered in the PROSPERO database (CRD42024615692).

## Key Points

Local tissue lengthening and muscle contraction increase the stiffness of remote structures within the same myofascial chain.

The certainty about the evidence is very low for lengthening and moderate for contraction.

Our findings open new frontiers for treatments of musculoskeletal pain, i.e., if local exercise is contraindicated.

## Background

Exercise is known to trigger a variety of adaptations on the tissue level, such as muscular hypertrophy or increased tendon cross-sectional area [1–3]. Even in the short term, mechanical loading substantially affects the soft tissue. A single bout of stretching can immediately reduce muscle stiffness, which is an often-sought-after goal in patients and athletes [4–6], e.g., for recovery after exercise sessions, or to alleviate excessive muscle tone, spasms and pain [7, 8]. Hitherto, most research addressing the acute impact of exercise on the musculoskeletal system has focused on muscles, tendons, and ligaments, but there is a paucity of studies examining the deep fascia.

Fascia is a multi-layered fibrous connective tissue that wraps around muscles and organs. It mainly consists of collagen and displays a rich sensory innervation [9]. Fascia, furthermore, has autonomous capacities for stiffness regulation: It can modify its viscoelastic properties via cellular contraction [10], fluctuation in the water content [11, 12], and thixotropy of the hyaluronic acid between the fascial layers [13]. Recently, a new role in joint stability has been discussed. According to modeling data, the thoracolumbar fascia is the primary passive stabilizer of the lower back, contributing more to spinal stability than muscle contraction or the generation of intraabdominal pressure [14]. Contrary to earlier assumptions, this all means that fascia is not a merely passive rope, but rather a mechanically adaptive tissue potentially receptive to exercise.

Besides its inert mechanical features, fascia links neighboring skeletal muscles, creating a body-wide network of myofascial chains [15, 16]. Interestingly, several studies demonstrated remote exercise effects following local exercise interventions [17]. Static stretching performed on the calf and dorsal thigh (two components of the posterior myofascial chain) enhances cervical range of motion (ROM) [18] and self-massage of the plantar fascia improves hamstring flexibility [19]. This effect may represent a response of the central nervous system as the application of a remote painful stimulus has been shown to trigger endogenous pain modulation and to increase non-local range of motion [20]. However, both biomechanical cadaver trials and in vivo experimental studies using high-resolution ultrasound imaging also verified a direct force transmission between neighboring skeletal muscles upon tissue lengthening [21, 22]. It has, therefore, been suggested to use distant exercise treatments to modify local tissue properties. This could be of interest (a) as a strategy to enhance the effect of local exercise or (b) if local exercise is contraindicated (e.g., in musculoskeletal disorders).

Despite the experimental verification of in-series myofascial force transmission [15] and the existence of functional remote exercise effects (i.e., increased ROM), there is no systematic investigation on whether local exercise based on myofascial chains can alter distant mechanical tissue properties. This systematic review with meta-analysis aimed to summarize the effects of local tissue lengthening and muscle contraction on the stiffness of remote structures within the same myofascial chain.

## Methods

A systematic review with meta-analysis was conducted in accordance with the PRISMA (Preferred Reporting Items for Systematic Reviews and Meta-Analyses) guidelines. It was prospectively registered in the PROSPERO database (CRD42024615692).

### Literature Search

Two independent researchers (JW, LK) performed a systematic literature search (November 2024). Relevant articles were retrieved from MEDLINE (PubMed), Web of Science, and Google Scholar. MEDLINE and Web of Science were chosen as they are among the databases providing most unique hits [23] and Google Scholar was added to complement the title and abstract searches of the two databases by an algorithm screening full texts.

As most previous studies examining non-local exercise effects and myofascial force transmission investigated the posterior myofascial chain (plantar aponeurosis, calf, dorsal thigh, lower back and back extensors), our search terms focused on the involved soft tissue components. The specific string was: (“knee flexors” OR “knee angle” OR “knee flexion” OR hamstrings OR “erector spinae” OR “lumbar” OR “thoracolumbar” OR “plantar fascia” OR “plantar aponeurosis” OR “plantar flexors” OR “triceps surae” OR calf OR gastrocnemius) AND (stiffness OR elasticity OR elastography OR ultrasound OR “elastic modulus” OR “passive torque”) AND (“force transmission” OR myofascial). For Google Scholar, we followed the procedure described by Wilke et al. [15] and screened the first 100 entries representing the most relevant hits. To supplement database searches, the reference lists of eligible papers were manually scanned [15].

Studies were considered eligible if meeting the following criteria: (1) controlled cross-sectional or controlled intervention (baseline data) trial design, (2) enrollment of healthy adults, (3) acute application of local tissue stretching or muscle contraction, (4) simultaneous assessment of tissue stiffness in a body region which is distant but structurally connected to the lengthened/contracted muscle within a myofascial chain, (5) publication in English or German in a peer-reviewed journal. Studies examining chronic effects or patients with diseases or pain conditions were excluded.

### Risk of Bias

Methodological study quality and risk of bias were assessed using an adapted version of the Downs and Black checklist, which has been demonstrated to exhibit high reliability and validity in the assessment of non-randomized studies [24]. Disagreements between the two independent raters (LK, JW) were resolved by discussion. To estimate the risk of a reporting bias, we evaluated the symmetry of funnel plots (effect sizes against standard errors) for dependent effect sizes. In cases of a minimal number of 10 studies, the Egger’s test was additionally used to statistically identify asymmetry [25]. The certainty about the evidence was classified as very low, low, moderate, or high, adhering to the criteria of the GRADE framework [26, 27]. The approach initially assumes a high certainty about the computed effect estimate, and in cases of risk of bias, imprecision, inconsistency, or indirectness, one level is subtracted per weakness. Conversely, a large effect magnitude leads to a higher level of certainty.

### Data Extraction

Two investigators (LK, JW) extracted data on sample size, participant characteristics, interventions, assessment methods, and study findings. Stiffness, the resistance of a tissue to mechanical deformation, represented the primary outcome. All data collected using valid measures (i.e., passive resistive torque or shear modulus) were included. For both the intervention (local stretch/contraction) and the control condition (no stretch/contraction), we obtained the means and standard deviations of tissue stiffness recorded simultaneously in a distant muscle located within the same myofascial chain. In cases of incomplete reporting (e.g., missing standard deviations), data were requested from the study authors, estimated from figures, p-values, t-values, standard errors, or imputed using the recommendations of the Cochrane handbook [27]. Where multiple comparison (e.g., stiffness in different joint angles or contraction intensities, multiple time points, multiple stiffness measurement methods) were available, all data were extracted.

### Data Synthesis and Statistics

Robust variance estimation with small sample size correction was applied to pool the standardized mean differences (SMD) and 95% confidence intervals (CI) between distant tissue stiffness measured during a local intervention (stretch/contraction) and an inactive control condition (local tissue inactive/in a slack position). Dependency of effect sizes (e.g. in cases of more than one stiffness measure or intervention condition) was considered by nesting the term ‘study’ as a random factor in the model. In addition, to account for dependency of repeated measures in crossover trials, a conservative correlation of *r* = 0.5 was assumed when calculating the effect estimate (Hedges' g) [28]. Summarized effect sizes were interpreted as small (SMD = 0.2 to 0.49), moderate (SMD = 0.50 to 0.79), or large (SMD = ≥ 0.8) and the significance level was set to alpha = 0.05. Heterogeneity between studies was quantified using Tau².

For outlier detection, we identified studies with absolute standardized residuals > 2. Sensitivity analyses leaving out studies with outliers were then performed to check the robustness of our findings [29]. To further assess potential statistical interferences caused through studies, which contributed a disproportionate number of effect sizes, we conducted a leave-one-out (LOO) sensitivity analysis, sequentially removing all effect sizes from a given study and re-estimating the model using RVE. Influence was quantified as the percentage change in the pooled estimate relative to the full model, with changes exceeding 10% flagged as potentially influential. A third set of sensitivity analyses were performed examining the impact of electromyography (EMG) use. Muscle activity, except for that produced locally in the contraction condition, may cause stiffness changes which are not related to force transmission mechanisms. Some of the studies included therefore used EMG to verify that the non-local muscles were inactive. In addition to a sensitivity analysis leaving out studies without EMG control, we performed a meta-regression with EMG use (yes/no) as a predictor variable. The software used was R (R Foundation for Statistical Computing, Vienna, Austria), with the packages metafor and robumeta for the meta-analysis, and dplyr for funnel plots.

## Results

### Search Results and Study Selection

**Fig. 1:**
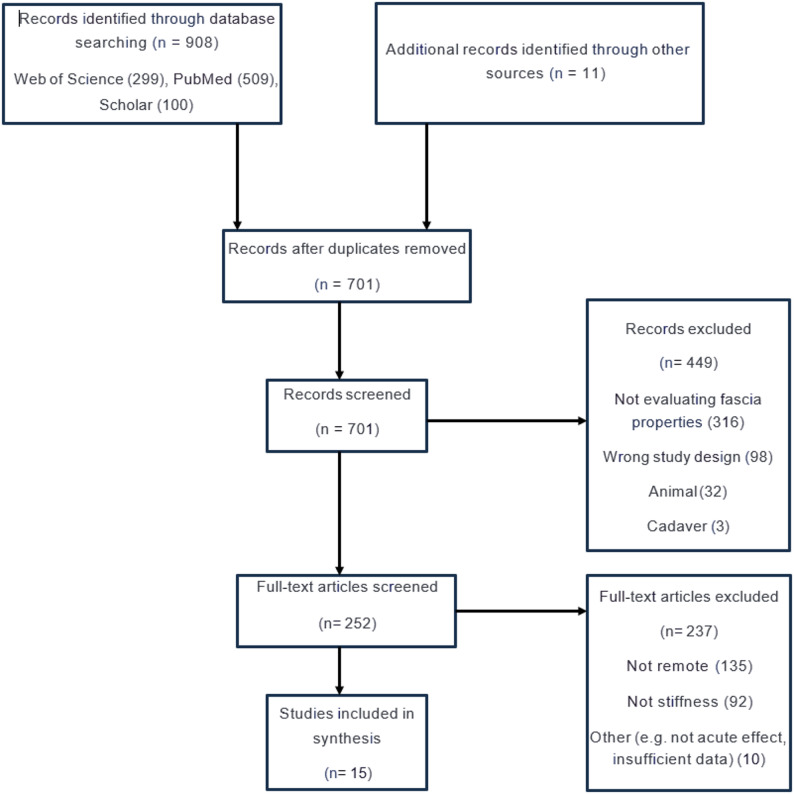
PRISMA chart displaying the flow of the literature search

The systematic searches identified a total of 908 studies, with an additional 11 records sourced from other references. After removal of duplicates and application of exclusion criteria, 15 studies were eligible for inclusion (Fig. 1).

### Study Characteristics

Collectively, the 15 papers (Table 1**)** examined 346 participants and of these, 216 were men and 130 were women. The mean age of the samples ranged from 18 ± 1 to 33 ± 7 years. Two from 15 studies recruited recreationally active runners and soccer players. The remaining 13 studies examined healthy participants without specific characteristics or conditions. Most studies (*n* = 13) used a crossover design while two adopted a parallel-group design. Twelve studies investigated the remote effects of local tissue lengthening/stretching, while seven trials evaluated the non-local impact of muscle contraction. Regarding stiffness measurements, eight trials used elastography, five trials used a dynamometer, and three studies used indentation-based methods. Fourteen studies performed measurements either during the contraction phase or, in the case of lengthening assessments, during the stretching. One study using dynamometers started the passive torque assessment immediately after the intervention condition. For details regarding timing of measurements, refer to Table 1.

**Table 1:** Characteristics of the studies included in the meta-analysis

Study	Participants	Design	Intervention	Measured structure/joint and Location	Measurement method	Outcome	EMG control
Caldeira et al. [30]	*n* = 54 (27 sedentary controls, 33.26 ± 7.39 years, 1.68 ± 0.09 m, 71.18 ± 13.71 kg, 14 females; 27 runners, 33.4 ± 7.1 years, 1.69 ± 0.09 m, 66.98 ± 9.69 kg, 14 females)	Parallel Group	*Contraction*: LD (prone, 10° shoulder extension, 10% MVIC)	TLF (2.5 cm laterally to L2/3) and Hip	Myotonometry (TLF), immediate measurement during Contraction Dynamometer (Hip), after one warm-up set immediate measurement in respective conditions	TLF: Stiffness (N/mm) Hip: passive torque (Nm)	Yes
Carvalhais et al. [31]	*n* = 37 (24.9 ± 3.2 years, 22 women), 1.69 ± 0.09 m, 64.43 ± 11.02 kg	Crossover	*Contraction*: LD (prone, shoulder position not specified, 25% MVIC) *Lengthening*: LD (prone, 120° shoulder flexion)	Hip	Dynamometer, after one warm-up set immediate measurement in respective conditions	Passive torque (Nm/rad)	Yes
Chen et al. [32]	*n* = 20 healthy females, 21.1 ± 1.7 years, 1.61 ± 0.04 m, 49.5 ± 5.1 kg	Crossover	*Contraction*: GA (prone, neutral ankle angle, 0%, 20%, 40%, 60% MVIC)	TLF (2 cm laterally to L2-3, L3-4 midline)	Shear-wave elastography, immediate measurement at reaching the testing MVICs	Stiffness (kPa)	No
Chen et al. [33]	*n* = 20 healthy males, 18.4 ± 0.7 years, 1.73 ± 0.05 m, 61.1 ± 9.6 kg	Crossover	*Lengthening*: HA (standing/sitting 30° and 60° hip flexion)	TLF (2 cm laterally to L2-3, L3-4 midline)	Shear-wave elastography, immediate measurement in respective conditions	Stiffness (kPa)	No
Chen et al. [34]	*n* = 20 healthy males, 18.4 ± 0.7 years, 1.73 ± 0.05 m, 61.1 ± 9.6 kg	Crossover	*Lengthening*: HA (sitting, 0° and 60° hip flexion)	TLF (2 cm laterally to L2-3, L3-4 midline)	Shear-wave elastography, immediate measurement in respective conditions	Stiffness (kPa)	No
Huang et al. [35]	*n* = 30 (15 males, 15 females), 22.9 ± 3.8 years, 1.65 ± 0.09 m	Crossover	*Lengthening*: HA (prone, 0° and 90°knee flexion)	PFA (posterior end of PFA between first and second metatarsal bone, in various ankle angles)	Myotonometry, immediate measurement in respective conditions	Stiffness (N/mm)	No
Kellis et al.[36]	*n* = 24 healthy males (13 without history of hamstring injury, 27.3 ± 4.6 years, 1.74 ± 0.05 m, 71 ± 4.3 kg and 11 with history of hamstring injury, 28.3 ± 6.7 years, 1.76 ± 0.05 m, 73.5 ± 8.3 kg)	Parallel Group	*Contraction* HA (prone, 0°, 45°, 90° knee flexion, submaximal contraction) *Lengthening*: HA (prone, 0° and 90° knee flexion)	TLF (2.5 cm laterally to L3/4)	Shear-wave elastography, after a two second force build up the measurements were taken during a 5 s hold.	Stiffness (kPa)	Yes
Kellis et al. [37]	*n* = 15 healthy males, 23.1 ± 3.2 years, 184 ± 0.05 m, 77.3 ± 4.11 kg	Crossover	*Contraction* HA (prone, 0°, 45°, 90° knee flexion, 70% MVIC) *Lengthening*: HA (prone, 0°, 45°, 90° knee flexion)	MF, TLF (4 cm laterally to L3/4), SM and ST	Shear-wave elastography, after a two second force build up the measurements were taken during a 5 s hold.	Stiffness (kPa)	Yes
Kellis et al. [38]	*n* = 14 healthy males, 23.7 ± 7.3 years, 1.84 ± 0.48 m, 77.3 ± 4.11 kg	Crossover	*Contraction* HA (prone, 0°, 45°, 90° knee flexion, submaximal contraction) *Lengthening*: HA (prone, 0°, 45°, 90°, knee flexion)	SM, ST, and TLF (2.5 cm laterally to L3/4)	Shear-wave elastography, after a two second force build up the measurements were taken during a 5 s hold.	Stiffness (kPa)	No
Liu et al. [39]	*n* = 20 healthy males, 21.3 ± 0.9 years, 1.71 ± 4.99 m, 61.02 ± 5.6 kg	Crossover	*Lengthening*: HA (prone, 0° and 90° knee flexion)	PFA (0, 3, 6 cm proximal to the calcaneal tuberosity, in various ankle angles)	Shear-wave elastography, immediate measurement in respective conditions, image acquisition 5–8 s	Stiffness (kPa)	No
Marinho et al. [40]	*n* = 37, 24 ± 3.5 years, 21 females, height and weight not reported	Crossover	*Lengthening*: HA (supine, 0° and 90° hip and knee flexion)	Ankle	Dynamometer, immediate measurements in testing positions	Passive torque (Nm)	Yes
Murakami et al. [41]	*n* = 18 healthy males, 21.9 ± 3.1 years,1.64 ± 0.23 m, 67.3 ± 12 kg	Crossover	*Lengthening*: HA (sitting, trunk flexion, angle not specified)	Ankle	Dynamometer, immediately after stretching intervention	Passive torque (Nm)	Yes
Palmer et al. [42]	*n* = 5 healthy males, 24 ± 3 years, 1.78 ± 0.06 m, 85 ± 10 kg	Crossover	*Lengthening*: GA (supine, DF, PF and neutral ankle angle)	Hip	Dynamometer, immediate measurements in testing positions	Passive torque (Nm)	Yes
Shiotani et al. [43]	*n* = 12, 23.4 ± 2.6 years, 1.68 ± 0.08 m, 59.5 ± 7.5 kg, 4 females	Crossover	*Lengthening*: GA (supine, 20° PF and 10° DF)	PFA (0, 3, 6 cm proximal to the calcaneal tuberosity)	Shear-wave elastography, immediate measurement in respective conditions within a seven second measurement window	Shear wave velocity (m/s)	Yes
Zhang et al. [44]	*n* = 20 healthy females, 20 ± 1 years, 1.60 ± 0.05 m, 50.75 ± 6.14 kg	Crossover	*Contraction*: ES (prone, trunk extension, angle not specified, 0%, 30%, and 60% MVIC)	Medial/lateral GA (30% of the line from medial/lateral popliteal stripe to the lateral ankle), ST (midpoint of sciatic tubercle to the medial tibial condyle), BF (the midpoint of sciatic tubercle to the lateral tibial condyle) and ES	Myotonometry, immediately during contraction but over a time span of 20–30 s multiple measurements was performed	Stiffness (N/mm)	No

Age data are means ± standard deviation

*MVIC* maximum voluntary isometric contractions, *HA* Hamstrings,* LD* Latissimus Dorsi, *GA* gastrocnemius, *EMG* electromyography, *PFA* plantar fascia, *PF* plantar flexion, *DF* dorsal flexion, *ST* semitendinosus, *SM* semimembranosus, *TLF *thoracolumbar fascia, *ES* erector spinae, *MF* multifidii

### Methodological Quality and Risk of Bias

The mean rating on the modified Downs and Black checklist was 11.33 ± 1.7 points (maximum score: 14). Most studies had clear reporting, samples representative of the target population, sufficient study power, valid measurement methods, no indication of data dredging, and reports of variability estimates as well as exact p values (Table 2). Weaknesses were a frequent lack of concealed allocation and a lack of randomization in the treatment sequence in crossover trials (8 of 13 trials). Another shortcoming was inadequate measurement and adjustment for confounders. Specifically, an important aspect in stiffness measurements and force transmission studies is controlling unwanted muscle activity. However, only eight out of 15 studies in our sample applied EMG to monitor unwanted muscle activity. Funnel plots showed considerable asymmetry for both lengthening and contraction which was mainly due to one outlier in each of the comparisons. The Egger’s test yielded a significant result for lengthening (*p*<0.01) while the number of studies (*n* = 7) was too small to perform the test for contraction.

**Table 2:** Methodological quality of the studies included (ratings on modified Downs and Black checklist)

	1	2	3	4	5	6	7	8	9	10	11	12	13	14	15
Caldeira et al. [30]	1	1	1	1	1	1	1	1	1	1	1	0	1	1	13
Carvalhais et al. [31]	1	1	1	1	1	0	1	1	1	1	1	0	1	1	12
Chen et al. [32]	1	1	0	0	1	1	0	0	0	1	1	0	0	1	7
Chen et al. [33]	1	1	1	0	1	1	1	1	1	1	1	0	0	1	11
Chen et al. [34]	1	1	1	0	1	0	0	1	1	1	1	0	0	1	9
Huang et al. [35]	1	1	1	0	1	1	1	0	1	1	1	0	0	1	12
Kellis et al.[36]	1	1	1	0	1	1	0	1	1	1	1	0	0	1	10
Kellis et al. [37]	1	1	1	1	1	1	1	0	1	1	1	0	1	1	12
Kellis et al. [38]	1	1	1	1	1	1	1	0	1	1	1	1	1	1	13
Liu et al. [39]	1	1	1	0	1	1	0	1	0	1	1	1	0	1	10
Marinho et al. [40]	1	1	1	1	1	0	1	1	1	1	1	0	1	1	12
Murakami et al. [41]	1	1	1	1	1	1	1	0	1	1	1	1	0	1	12
Palmer et al. [42]	1	1	1	1	1	1	1	0	1	1	1	1	1	1	13
Shiotani et al. [43]	1	1	1	1	1	1	1	1	1	1	1	0	1	1	13
Zhang et al. [44]	1	1	1	0	1	1	1	0	1	1	1	1	0	1	11

1 point awarded, 0 no point awarded; 1=Aim, 2=Outcomes, 3=Sample, 4=Confounder, 5=Findings, 6=Variability, 7=p-value, 8=Subjects, 9=Dredging, 10=Statistics, 11=Accurate Outcome, 12=Randomised, 13=Confounding, 14=Gpower, 15=Total

### Effects of Lengthening and Contraction on Non-local Stiffness

**Fig. 2:**
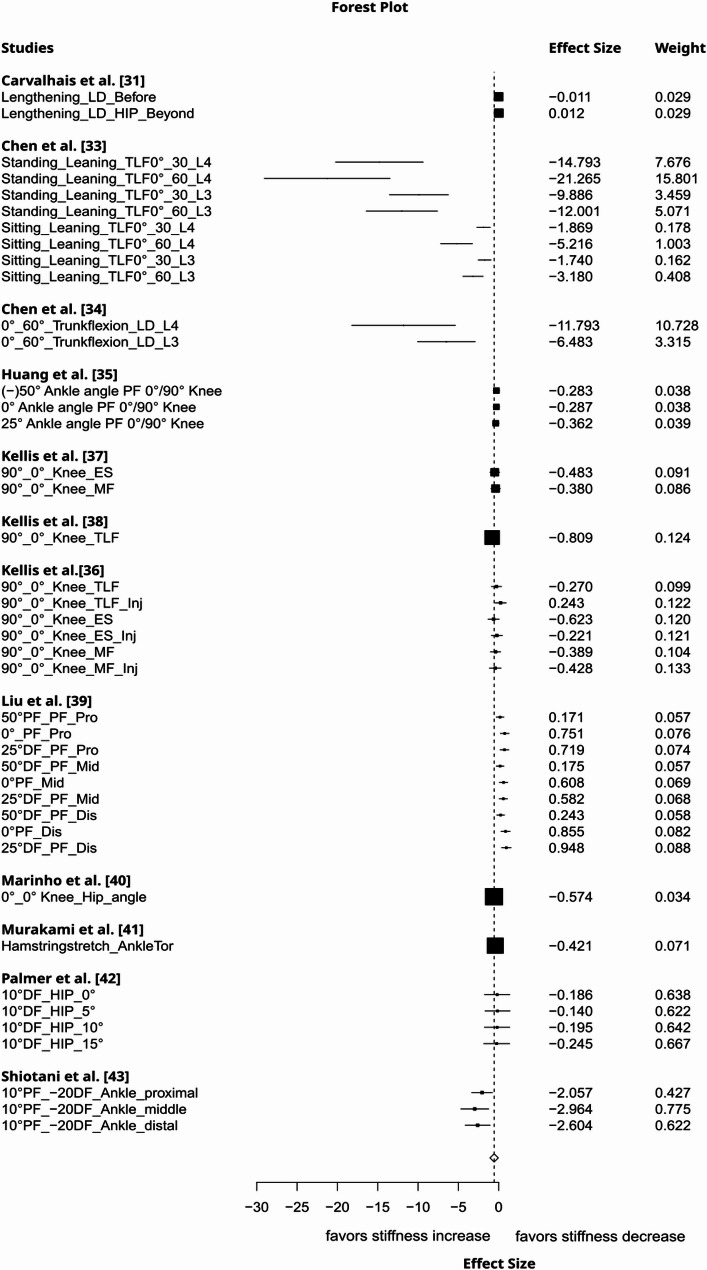
Forest plot displaying the effect of local tissue lengthening on remote tissue stiffness with effects and weight. An additional description of the intervention and measured structure is provided beneath the study names

**Fig. 3:**
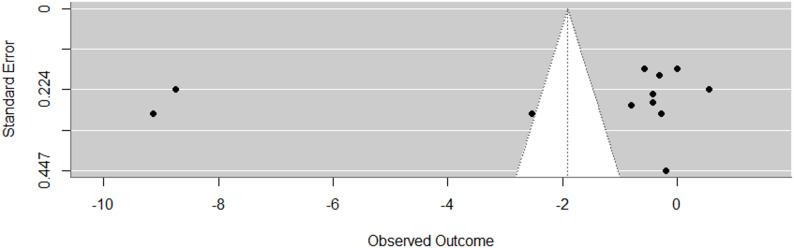
Funnel plot of the overall effect of local tissue lengthening on remote tissue stiffness (effect sizes against standard error)

**Fig. 4:**
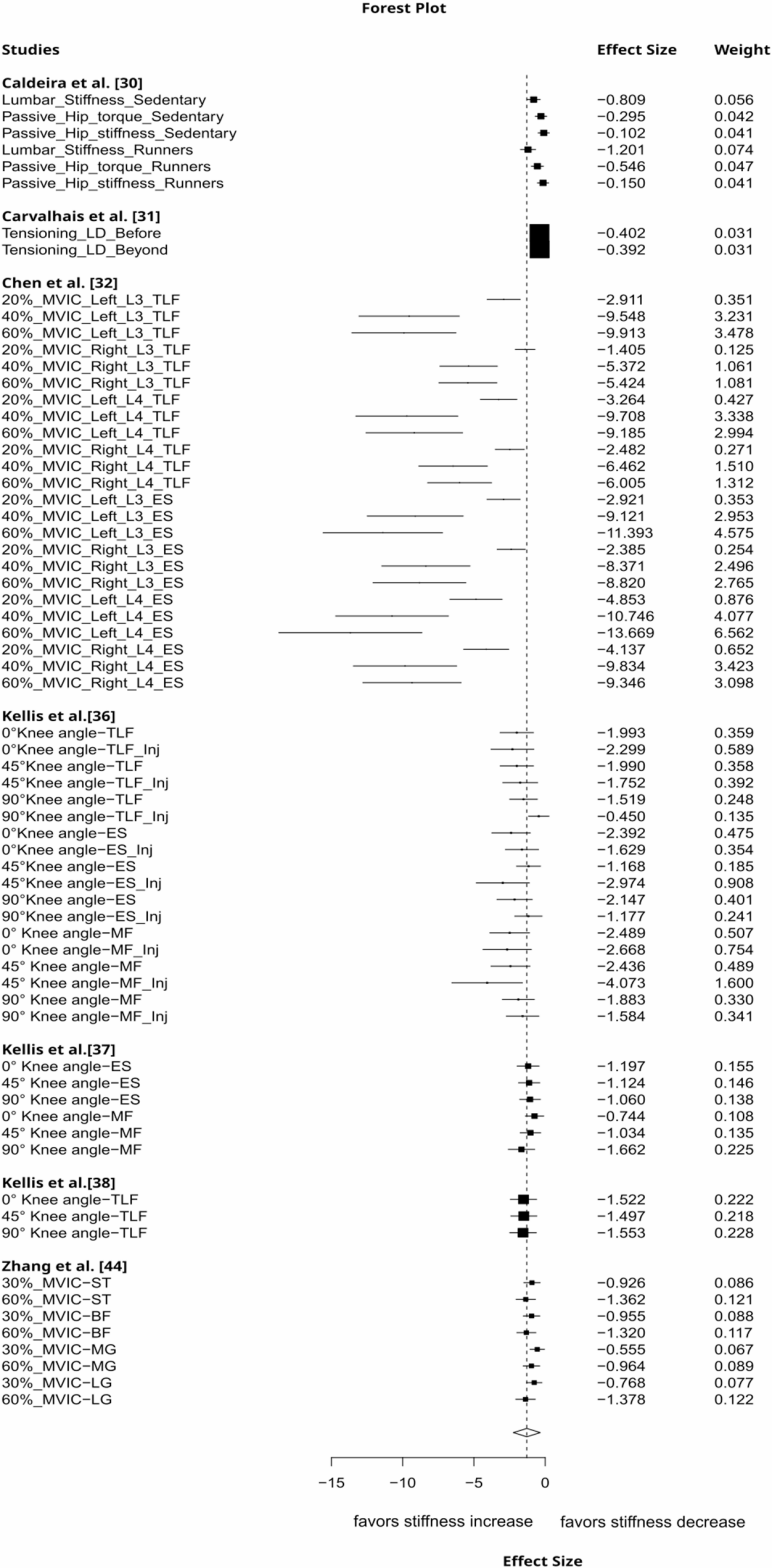
Forest plot displaying the effect of local muscle contraction on remote tissue stiffness with effects and weight. An additional description of the intervention and measured structure is provided beneath the study names

**Fig. 5:**
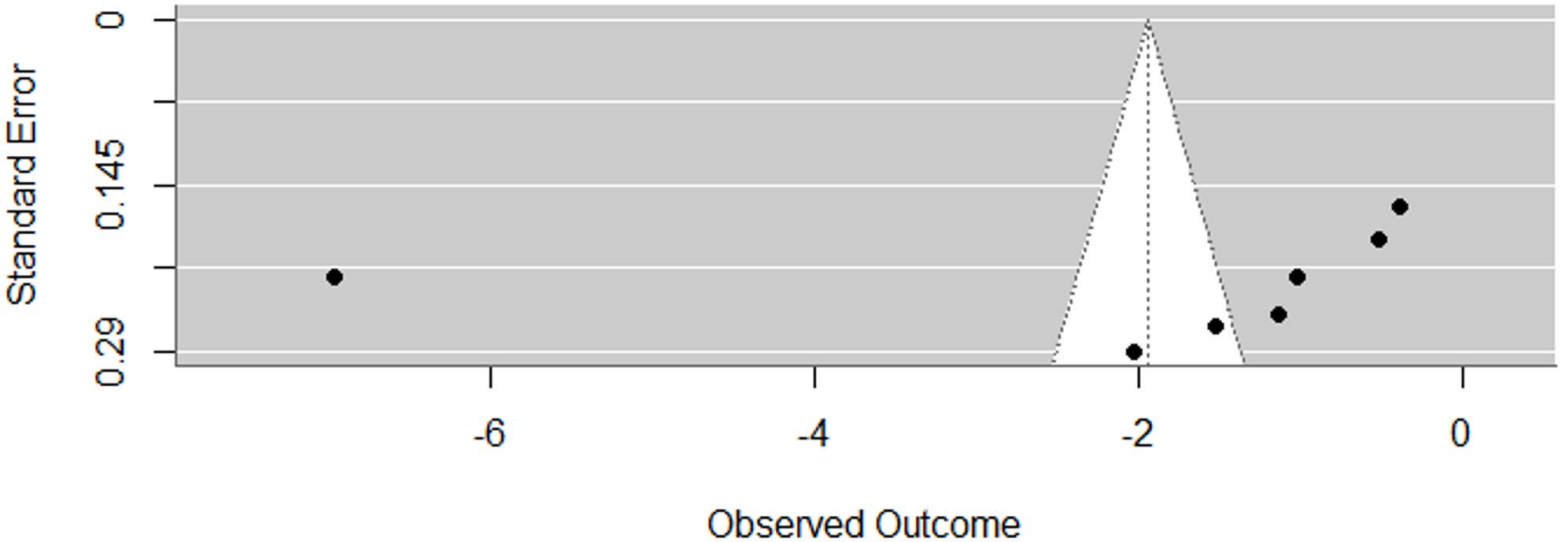
Funnel plot of the overall effect of local muscle contraction on remote tissue stiffness (effect sizes against standard error)

Local lengthening increased remote tissue stiffness to a moderate degree (g= −0.54, 95%CI: −1.03 to −0.05, *p* = 0.04, 12 studies, 42 effect sizes (ES), τ² = 0.42, Fig. 2). The certainty about the evidence was rated as very low due to imprecision (lower bound of 95% CI included a trivial effect size), inconsistency (substantial between-study heterogeneity), and risk of publication bias (funnel plot asymmetry, Fig. 3). Muscle contraction induced a large increase in non-local stiffness (g= −1.25, 95%CI: −2.15 to −0.34, *p* = 0.016, 7 studies, 67 ES, τ² = 0.61, Fig. 4). The certainty about the evidence was rated as moderate (downrank for inconsistency/substantial between-study heterogeneity and risk of publication bias (Fig. 5), up rank for large-magnitude effect).

**Fig. 6:**
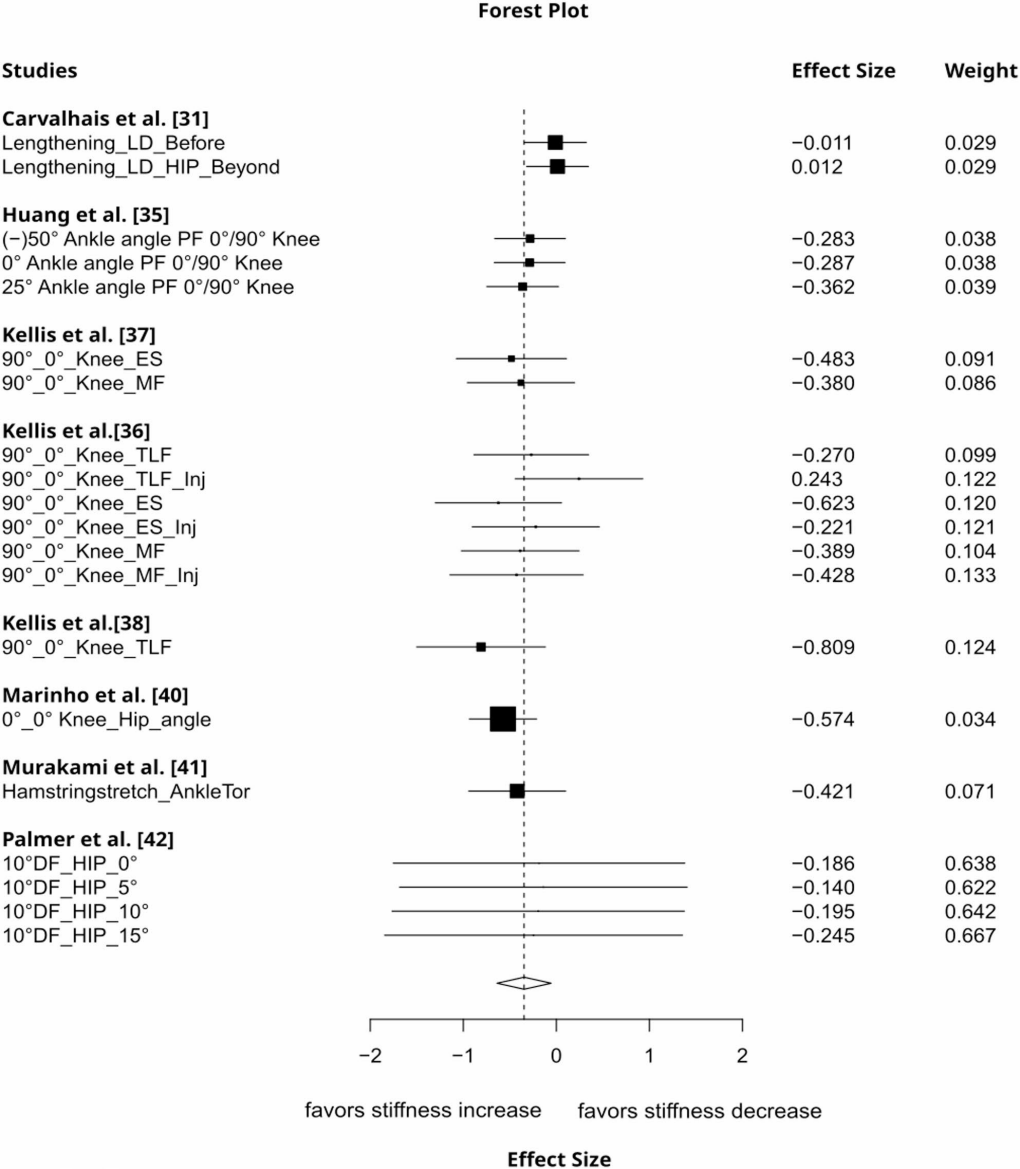
Forest plot displaying the effect of local tissue lengthening (outliers excluded) on remote tissue stiffness with effects and weight. An additional description of the intervention and measured structure is provided beneath the study names

**Fig. 7:**
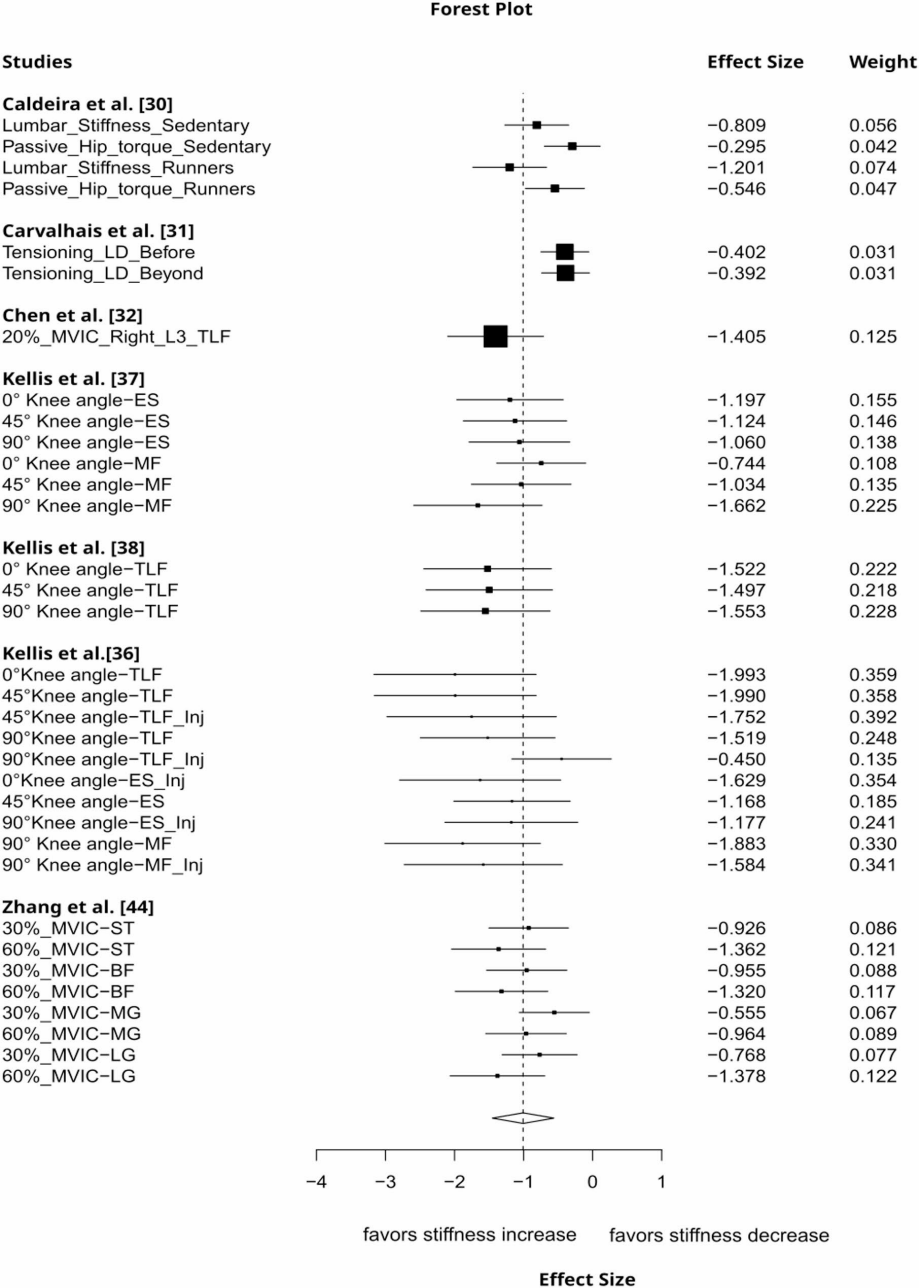
Forest plot displaying the effect of local muscle contraction (outliers excluded) on remote tissue stiffness with effects and weight. An additional description of the intervention and measured structure is provided beneath the study names

Removing outliers did not alter the outcome of both meta-analyses meaningfully and even with extreme values removed, the observed effects were significant and non-trivial (lengthening: g= −0.34, *p* = 0.03, 95% CI −0.63 to −0.05, 8 studies with 20 ES, Fig. 6, contraction: g= −0.98, *p* < 0.01, 95%CI −1.41 to −0.55, 7 studies with 34 ES, Fig. 7). Furthermore, the study-level leave-one-out sensitivity analysis for lengthening revealed that effect estimates ranged from b = −0.413 to b = − 0.269, with all iterations remaining statistically significant (all *p* < 0.05). The most influential study was Carvalhais et al. [31] (k = 2 effect sizes, Δb = − 0.089, − 27.6%), while the least impactful was Kellis et al. [37] (Δb = + 0.002, + 0.5%). Notably, Kellis et al. [36], which contributed the largest number of effect sizes (k = 6), exerted minimal influence on the pooled estimate (Δb = − 0.006, − 1.9%). Across the contraction LOO analysis, estimates ranged from b = − 1.106 to b = − 0.918, and all iterations remained statistically significant (all *p* < 0.01). In contrast Zhang et al. [44] (k = 8, Δb = − 0.006, − 0.6%) had the smallest effect. Kellis et al. [36], despite contributing the highest number of effect sizes (k = 10), produced only a modest change upon exclusion (Δb = + 0.050, + 5.1%). Therefore, both LOO analyses support the robustness of the findings.

The sensitivity analyses removing studies without EMG yielded slightly smaller effect sizes with limited power due to the smaller sample size (lengthening: g= −0.82, *p* = 0.14, 95% CI −1.29 to 0.3, 5 studies with 11 ES, contraction: g= −0.82, *p* = 0.07, 95% CI −1.76 to 0.3, 4 studies with 32 ES). Meta-regression using EMG control as a predictor provided no indication of a confounding effect (lengthening: *p*=0.82, contraction: *p*=0.36).

## Discussion

To the best of our knowledge, this is the first systematic review to investigate the effects of local tissue lengthening and muscle contraction on the mechanical properties of remote structures within the same myofascial chain. Based on our analysis of 15 studies, there is very low-certainty evidence for a moderate non-local stiffness increase following lengthening and moderate-certainty evidence for a large non-local stiffness increase following contraction.

These findings seemingly support previous works demonstrating in-series force transmission between skeletal muscles in cadavers and humans [15, 45, 46].

At first glance, a non-local stiffening upon lengthening contradicts previous reports of non-local ROM improvements following stretching or foam rolling [47–49]. This is because enhanced ROM, besides other factors, is typically associated with decreased but not increased stiffness [50]. However, the trials included in this systematic review measured simultaneous stiffness changes in the moment of local contraction and lengthening (see Table 1). From a physiological point of view, stretching causes a transient increase in tension in the moment of being applied, which is only followed by a later decrease (i.e., after stretching cessation). The underlying mechanism is based on the viscoelastic behavior of the soft tissue which displays a stress-relaxation phenomenon when being loaded [51, 52]. The same (increase of stiffness during exercise and a decrease in stiffness after exercise) may apply to non-local effects but should be verified by future studies.

The practical implications of our findings primarily apply to healthy population such as athletes. Increasing non-local stiffness during stretch or contraction within a myofascial chain may represent a strategy of the body to generate higher levels of stability in the respective joints. Increasing stiffness to an optimal degree will help absorb excessive forces, e.g. during eccentric loading, and thus to improve movement safety [53]. Non-local stiffness increases could also be beneficial for strength and performance. Kalkhoven & Watsford (2017) demonstrated positive associations between muscle stiffness and a variety of related markers in soccer players [54]. Finally, stiffness represents a significant contributor to movement efficiency as several trials demonstrated a link between a stiffer myo-tendinous complex and functional outcomes such as running economy [55]. In addition to the value of non-local stiffness increases for healthy populations, a perspective for future research is in the rehabilitation context. Altered tissue stiffness has been associated with various entities of musculoskeletal pain although there is some heterogeneity depending on pathology and affected body regions [56]. Patients often express uncertainty about movement and exercise in painful areas, and physiotherapists or physicians may rule out local treatment [57]. The possibility of altering the biomechanical properties of the soft tissue without directly targeting the affected body region offers an innovative and feasible approach to enhancing treatment effectiveness and to fostering patient activity instead of advising inactivity.

Some methodological aspects warrant consideration. In the contraction condition, studies used varying contraction intensities and protocols, which may influence the magnitude of the effect estimates. Regarding the outcome, tissue stiffness is not a constant, neither temporally, nor locally. Previous trials demonstrated site-dependent variations in hamstring, soleus, and gastrocnemius stiffness, with values being higher distally than proximally [58, 59]. This means that the area to be chosen for measurements is of importance. In a similar vein, stretching can have non-homogenous effects in the targeted muscle [60]. In our meta-analysis, most studies using elastography had multiple regions of interest within one image, in order to control for basic stiffness variations. However, they did not use multiple transducer locations (e.g., local and distal). It is unknown if the local variability in stretching effects also occurs non-locally. Notwithstanding, future studies should incorporate multiple locations to adequately consider this phenomenon and prevent bias induced by measurement sites.

It is tenable to ascribe non-local stiffness increases to the concept of myofascial chains. Yet exercise acts on the central nervous system in several ways. For instance, stretching alters pain perception via conditioned pain modulation [20]. Although this effect typically results in lower pain sensitivity, stretching treatments, in some cases, may also trigger protective muscle activity and stiffness increases. Second, neuromuscular inhibition has been discussed in the context of stretching treatments, causing a transient H-reflex reduction [61]. However, as the present meta-analysis found increased but not decreased stiffness after non-local stretching [62], inhibition does not seem to have played a significant role.

Certainty according to the GRADE criteria was very low for local lengthening. The examined studies used a variety of stiffness measurements, including elastography, indentation-based methods, and passive resistive torque derived from dynamometer data, and this may explain the high degree of heterogeneity. As, furthermore, the confidence interval of the effect estimate was wide, suggesting imprecision, and as reporting bias was suspected, additional confirmatory studies are needed before definitive conclusions on non-local stiffness changes following lengthening can be drawn.

Another caveat of the analyzed study sample is that only 8 out of 15 studies used EMG to control unwanted muscle activity. This is a critical design issue, as stiffness does not only increase if passive force is transmitted but also during active muscle contraction. We made a strong effort to investigate the possible impact of EMG use. On the one hand, sensitivity analysis could not fully rule out an impact of lacking EMG control. On the other hand, however, its interpretability was limited due to the small sub-sample size. Furthermore, as the meta-regression using EMG recordings as a factor provided no indications of a meaningful effect, we consider the potential confounding effect of not using EMG as small. Finally, 13 studies examined the structures of the posterior myofascial chain (plantar aponeurosis, Achilles tendon, calf, hamstrings, lumbar fascia, erector spinae), while the diagonal dorsal chain (gluteus maximus, lumbar fascia, contralateral latissimus dorsi), was investigated by two studies. Yet, there is solid evidence for the diagonal frontal chain [15] and a variety of arm chains [16], and hence, it would be prudent to extend the focus of biomechanical studies to other constituents of the myofascial network.

## Conclusion

There is moderate-certainty evidence that local contraction increases the stiffness of distant structures within the same myofascial chain. The same may occur during lengthening, but the certainty about the evidence is very low. Our findings are of relevance for coaches and therapists aiming to modify the mechanical tissue properties, e.g., when aiming to enhance performance or treat myofascial pain. Yet, in view of the still small number of available studies, methodological concerns in experimental conditions, and suspected reporting bias, additional research is needed before definitive conclusions on the relevance of non-local stiffness increases during local activity within myofascial chains can be drawn.

## Data Availability

The datasets generated and/or analysed during the current study are available from the corresponding author on reasonable request.
